# Dissolvable hyaluronic acid microneedles loaded with β-Elemene for the treatment of psoriasis

**DOI:** 10.3389/fphar.2022.1067051

**Published:** 2022-11-18

**Authors:** Chun Wang, Ruiqi Hao, Baowei Peng, Jiang Chang, Shisheng Chen, Yanxin Chen, Xiaohang Yin, Yumei Que, Chen Fan, Yuhong Xu

**Affiliations:** ^1^ College of Pharmaceutical Science, Dali University, Dali, China; ^2^ Joint Centre of Translational Medicine, The First Affiliated Hospital of Wenzhou Medical University, Wenzhou, China; ^3^ Wenzhou Institute, University of Chinese Academy of Sciences, Wenzhou, China; ^4^ Department of Dermatology, The Second Affiliated Hospital and Yuying Children’s Hospital of Wenzhou Medical University, Wenzhou, China

**Keywords:** β-Elemene, Psoriasis, Microneedles, apoptosis, inflammation

## Abstract

The pathology of psoriasis involves the over-proliferation of keratinocytes, exaggerated inflammation of keratinocytes, and infiltration of inflammatory cells such as macrophages (Mø), *etc.* The therapeutic outcomes of current treatment targeting one single pathological process are less than satisfactory. Based on their diverse biological activities, natural products offer a potential solution to this problem. In this study, we investigated the effects of β-Elemene (ELE) on both psoriatic keratinocytes and M1-type Mø (M1-Mø) *in vitro*. Hyaluronic acid (HA) microneedles loaded with ELE (HA-ELE-MN) were also fabricated and tested for the treatment of psoriasis *in vivo* using an imiquimod (IMQ)-induced psoriatic mice model. Our data suggest that ELE induces apoptosis and inhibits inflammation of psoriatic keratinocytes. In addition, ELE attenuates the expression of inflammatory cytokines secreted from M1-Mø, thus indirectly inhibiting the inflammation of keratinocytes. Furthermore, HA-ELE-MN has been found to significantly alleviate symptoms in an IMQ-induced psoriatic mice model by inducing keratinocytes apoptosis, suppressing keratinocytes proliferation, and inhibiting M1-Mø infiltration. Taken together, this study demonstrates that ELE can be used for the treatment of psoriasis by targeting both keratinocytes and M1-Mø, which provides a potential novel reagent for psoriasis treatment.

## 1 Introduction

Psoriasis is a chronic inflammatory skin disease in clinic, it has been reported that more than 120 million people suffer from psoriasis worldwide (Greb et al., 2016). It not only causes physical damage such as pain and itching (Dubertret et al., 2010) but also has a great negative impact on the mental health of the patients (Boehncke and Schon, 2015). In addition, studies also found that psoriatic patients have significantly increased risks of other chronic and serious complications, including psoriatic arthritis, metabolic syndrome, cardiovascular diseases, *etc.* (Barker Jonathan, 2007; Nestle et al., 2009). To date, the exact pathogenesis of psoriasis is still not fully understood. The over-proliferation and exaggerated inflammation of keratinocytes are the main pathological features of psoriasis (Zhou et al., 2022). Except for the environmental and genetic factors, excessive aggregation of inflammatory cytokines caused by the infiltration of various inflammatory cells is one of the main causes of the over-proliferation and inflammation of keratinocytes. For example, studies have found that the number of CD68^+^ M1-Mø is significantly elevated in psoriasis skin, indicating that infiltration of M1-Mø plays an essential role in psoriasis (Kim et al., 2019). Further study revealed that infiltration of M1-Mø leads to an increased expression of various inflammatory cytokines such as tumor necrosis factor-α (TNF-α), interleukin-1 (IL-1), IL-6, IL-12, IL-23, and Interferon γ (IFN-γ). These inflammatory cytokines further stimulate keratinocytes proliferation and inflammation, thus leading to the onset of psoriasis.

Although there are various therapies for psoriasis in clinic, their therapeutic effects are limited and often cause various side effects. For example, methotrexate is a commonly used psoriasis drug by inducing apoptosis in keratinocytes (Elango et al., 2017), however, it has strong hepatotoxicity. Light therapy is also frequently used for the treatment of severe psoriasis by triggering keratinocytes apoptosis, while it causes erythema, itching, blistering, and photoaging (Armstrong and Read, 2020). In addition, corticosteroids have been demonstrated to relieve psoriasis by suppressing inflammation, but it is only suitable for mild psoriasis. The long-term use of corticosteroids results in skin atrophy, telangiectasia, and skin striae (Armstrong and Read, 2020). In recent years, various biologics targeting specific inflammatory cytokines, such as IL-17A inhibitors, TNF-α inhibitors, IL-23 inhibitors, *etc.*, have also been shown to be effective in treating psoriasis. However, side effects such as upper respiratory tract infections are observed after their clinical use (Armstrong and Read, 2020; Diotallevi et al., 2022). Therefore, the therapeutic effects of treatment targeting one single pathological process of psoriasis are unsatisfactory. With the increased understanding of the roles of inflammatory cells in psoriasis, recent studies also explored the possibility of treating psoriasis by regulating inflammatory cells such as Mø, however, the therapeutic effects need to be further evaluated (Tao et al., 2022). To improve the therapeutic effects, other studies also used combination therapies. For example, Wang et al. (2022) explored a chemo-photodynamic strategy using the combination of methotrexate and 5-aminolevulinic acid, which has been shown to effectively alleviate psoriasis by inducing apoptosis and inhibiting inflammation simultaneously (Wang et al., 2022). Although combination therapies have been found to generate better therapeutic outcomes, they make the pharmacological mechanisms more elusive, and the safety of these combination therapies needs to be further evaluated (Nakamura and Koo, 2020).

Natural products offer a potential solution to the above issue, as they often possess diverse biological activities, allowing the use of one molecule to simultaneously regulate multiple pathological processes. By analyzing the literature, we found that ELE holds both apoptosis-inducing and inflammation-inhibiting properties. For example, ELE has been shown to induce apoptosis in various tumor cell lines including hepatocellular carcinoma cell lines (Wu et al., 2016), Osteosarcoma MG 63 and U2OS cell lines (Ding et al., 2018), and human non-small lung cancer A549 cell lines (Liu et al., 2012). In addition, Hu et al. (2020) found that ELE significantly inhibits the inflammatory responses in mouse skin tumor tissues (Hu et al., 2020). Han et al. (2021) reported that ELE attenuates the elevated expression of IL-1 in Lipopolysaccharides (LPS)-stimulated Mø, suggesting the inflammation-inhibiting ability of ELE by regulating Mø (Han et al., 2021). Based on the bioactivities of ELE, we therefore hypothesized that ELE induces apoptosis in psoriatic keratinocytes and inhibits inflammation in both psoriatic keratinocytes and M1-Mø, thus can be used for the treatment of psoriasis.

Since the epidermis layer of psoriatic skin is significantly thicker than that in normal skin, the effective delivery of drugs is therefore another key point for psoriasis treatment. To date, most of the anti-psoriasis drugs are topically applied to the lesion by plasters, however, the poor drug delivery efficiency causes limited therapeutic effects (Qu et al., 2022). Particularly for natural products, although promising therapeutic effects can be observed in *vitro* studies, the therapeutic outcomes *in vivo* are often significantly reduced due to their unstable physicochemical properties, poor solubility, and low bioavailability. The emergence of microneedle (MN) technology offers a potential solution to this problem. Compared to traditional formulations, MN delivery significantly increases the drug concentration at the site of the lesion. In addition, MN transdermal delivery technique has been demonstrated to minimize systemic toxicity, sense of pain, and tissue invasion (Qu et al., 2022).

In this study reported herein, we investigated the effects of ELE on both psoriatic keratinocytes and M1-Mø. In addition, how ELE directly affects keratinocytes *via* the regulation of M1-Mø was also detected. Furthermore, the therapeutic effects of HA-ELE-MN were validated using an IMQ-induced psoriatic mice model.

## 2 Materials and methods

### 2.1 Experimental reagents

ELE was purchased from Macklin (Shanghai, China) and dissolved in DMSO to make a stock solution at 10 mM. IMQ was purchased from Med-Shine Pharmaceutical (Sichuan, China). TNF-α, IL-1α, IL-17, IL-22, and oncostatin-M (OSM) were purchased from Beyotime (Shanghai, China) and mixed together to make an M5 solution at 2.5 ng/ml based on the previously published method (Gao et al., 2020). LPS was purchased from Sigma (Shanghai, China) and dissolved in the cell culture medium to make a 10 ng/ml stock solution. Recombinant mouse IFN-γ was purchased from Novoprotein (Suzhou, China) and dissolved in sterile water to obtain a 10 ng/ml stock solution.

### 2.2 Cell culture

Human keratinocytes (HaCaT), dermal fibroblasts (HFF-1), and mouse Mø (RAW264.7) cell lines were purchased from American type culture collection (ATCC) and cultured in high glucose Dulbecco’s Modified Eagle’s Medium (DMEM) containing 10% fetal bovine serum (FBS) and 1% penicillin-streptomycin. Cells were maintained at 37 °C in an incubator with 5% CO_2_.

### 2.3 Cell viability

Effects of ELE on the viability of keratinocytes, fibroblasts, and M5-stimulated keratinocytes were measured using Cell Counting Kit (CCK-8; Yeasen Biotechnology, Shanghai, China). Briefly, the cells were seeded in a 96-well plate at 5000 cells/well for 24 h. Then different concentrations (50 and 100 μM) of ELE were applied to the cells for 72 h. To detect the effect of ELE on M5-stimulated keratinocytes, cells were seeded in a 96-well plate at 5000 cells/well for 24 h. Then the cells were further treated with ELE (100 μM) mixed with M5 (2.5 ng/ml) for 72 h. A group containing M5 only was also included as a control. Cell viability was measured by reading the absorbance at 450 nm after incubated with CCK-8 reagents for 1 h.

### 2.4 Cell apoptosis

Cell apoptosis triggered by ELE in M5-stimulated keratinocytes was detected using a TUNEL apoptosis assay kit (Beyotime). Briefly, keratinocytes (2×10^4^ cells/well) were seeded in a 48-well plate for 24 h and then treated with M5 mixed with ELE as described above. After exposure for 48 h, the cells were fixed and permeabilized using reagents provided in the assay kit. The cells were then further incubated with a biotin-labeled solution for 60 min followed by Streptavidin-HRP for another 30 min. Apoptotic cells were visualized using an Olympus CKX53 microscope (Olympus, Japan) after incubation with DAB chromogenic solution.

#### 2.5 RT-PCR

Keratinocytes (2×10^4^ cells/well) were seeded in a 48-well plate for 24 h, then the cells were treated with M5 mixed with ELE as described above for 48 h. For the Mø study, the cells were first resuspended in a medium containing LPS (100 ng/ml), IFN-γ (20 ng/ml), and ELE (100 μM), respectively. The cells (2×10^5^ cells/well) were then further seeded in a 48-well plate for 48 h. To identify the effects of ELE-pretreated M1-Mø, Mø was cultured in a medium containing LPS/IFN-γ or LPS/IFN-γ/ELE for 24 h. The medium was then discarded and replaced with fresh medium (without LPS/IFN-γ and ELE) for another 24 h. The conditioned medium (CM) was eventually collected and placed in dishes pre-seeded with keratinocytes for 48 h. Total RNA was extracted using MolPure Cell/Tissue miRNA Kit (Yeasen Biotechnology). The first strand cDNA was synthesized using HifairⅡ1st Strand cDNA Synthesis SuperMix for qPCR (Yeasen Biotechnology). qRT-PCR was performed using SYBR green reagent in an LightCycler 480 II (Roche, Swiss). Primers of targeted genes are listed in Table 1 below:

**TABLE 1:** Primers used in RT-PCR.

Protein Name	Corresponding Gene Name	Primer Sequences
For keratinocytes		
GAPDH	*GAPDH*	F: 5′-CAG​GAG​AGT​GTT​TCC​TCG​TCC-3′
		R: 5′-TTT​GCC​GTG​AGT​GGA​GTC​AT-3′
IL-1α	*IL1A*	F: 5′-CAT​GTC​AAA​TTT​CAC​TGC​TTC​ATC​C-3′
		R: 5′-GTC​TCT​GAA​TCA​GAA​ATC​CTT​CTA​TC-3′
IL-1β	*IL1B*	F: 5′-ATG​ATG​GCT​TAT​TAC​AGT​GGC​AA-3′
		R: 5′-GTC​GGA​GAT​TCG​TAG​CTG​GA-3′
IL-6	*IL6*	F: 5′-TCC​CAC​GAA​ATC​CAG​GAT​GC-3′
		R: 5′-GGA​TGT​TCA​GGT​TGA​CCA​TCA​C-3′
IL-8	*IL8*	F: 5′-ACT​GAG​AGT​GAT​TGA​GAG​TGG​AC-3′
		R: 5′-AAC​CCT​CTG​CAC​CCA​GTT​TTC-3′
Keratin 6	*KRT6*	F: 5′-GGG​TTT​CAG​TGC​CAA​CTC​AG-3′
		R: 5′-CCA​GGC​CAT​ACA​GAC​TGC​GG-3′
Filaggrin	*FLG*	F: 5′-TTT​CGT​GTT​TGT​CTG​CTT​GC-3′
		R: 5′-CTG​GAC​ACT​CAG​GTT​CCC​AT-3′
For Mø		
GAPDH	*Gapdh*	F: 5′-CAG​GAG​AGT​GTT​TCC​TCG​TCC-3′
		R: 5′-TTT​GCC​GTG​AGT​GGA​GTC​AT-3′
TNF-α	*Tnf*	F: 5′-TAG​CCC​ACG​TCG​TAG​CAA​AC-3′
		R: 5′-GCA​GCC​TTG​TCC​CTT​GAA​GA-3′
IL-1α	*Il1a*	F: 5′-GTC​GGG​AGG​AGA​CGA​CTC​TAA-3′
		R: 5′-GTT​TCT​GGC​AAC​TCC​TTC​AGC-3′
IL-12A	*Il12a*	F: 5′-TGT​GTC​AAT​CAC​GCT​ACC​TCC-3′
		R: 5′-TGG​TCT​TCA​GCA​GGT​TTC​GG-3′

### 2.6 HA-ELE-MN preparation

Polydimethylsiloxane (PDMS) mold was purchased from Taizhou Microchip Pharmaceutical Technology (Taizhou, China). The parameters of the PDMS mold are: needle length = 1 mm, bottom dimension = 0.45 mm * 0.45 mm, number of arrays = 15 * 15, and groove depth = 16 mm * 16 mm. HA powder was dissolved in deionized water to make a 7% (w/v) HA solution, and then the ELE stock solution (10 mM) was mixed with 7% HA solution at different ratios for MN containing different amounts of ELE. The HA-ELE mixtures were then poured into a PDMS mold, centrifuged at 4000 rpm for 5 min, and eventually dried in a fume hood at room temperature for 2 days. ELE-HA-MN was obtained by peeling off the PDMS mold and stocked at room temperature for future use.

### 2.7 Mechanical performance of HA-ELE-MN

As described above, the ELE stock solution was mixed with 7% HA solution at ratios of 1:250, 1:50, and 1:10 to make the concentration of ELE in ELE-HA-MN at 40, 200, and 1000 μM, respectively. The mechanical property of HA-ELE-MN was tested using Electronic universal Material Testing Machine (Instron 5944). In brief, MNs were placed on the metal platform with tips facing upwards. The cylindrical probe sensor was set to move down at the speed of 1 mm/s to compress the MNs. Force and displacement data were recorded and eventually processed into a force-displacement curve.

### 2.8 *In vivo* penetration of HA-ELE-MN

ELE-HA-MN (ELE concentration at 1 mM) was inserted into the back skin of normal BALB/c mice and IMQ-induced psoriasis BALB/c mice for 1 min, respectively. The skin was then removed from the mice, fixed with 4% paraformaldehyde, embedded, sectioned, and stained with Hematoxylin and Eosin. Penetration of skin was recorded using an Olympus CKX53 microscope (Olympus).

### 2.9 *In vivo* dissolution of HA-ELE-MN

ELE-HA-MN (ELE concentration at 1 mM) was inserted into the back skin of BALB/c mice for 0, 3, 5, and 10 min, respectively. The MNs were then peeled off and cut into strips. The dissolution of the tips was captured using an Olympus SZ61 microscope (Olympus).

### 2.10 IMQ-induced psoriasis mouse model establishment and treatment

The hair on the back skin of BALB/c mice (male, 8 weeks) was removed using a razor. After isoflurane inhalation anesthesia, the mice received topical treatment of 70 mg 5% IMQ cream on the back skin every morning for 7 days. Psoriasis-like symptoms including erythema and scales could be observed 3 days later. From then, HA-MN, HA-ELE-MN (1mM), and ELE Vaseline cream (1 mM) were applied every night for 4 days. Images of back skin were recorded every morning for the assessment of the psoriasis area and severity index (PASI). Parameters of PASI, including erythema, scales, and infiltration, were scored as follows: 0 (none), 1 (mild), 2 (moderate), 3 (severe), and 4 (very severe). These three parameter scores were then summed to obtain a total score that reflects the severity of inflammation in mice back. After stimulated with IMQ for 7 days along with the treatment of ELE for 4 days, the mice were sacrificed to obtain skin tissues for further analysis.

### 2.11 Histological analysis

The skin samples collected from the sacrificed mice were fixed with paraformaldehyde, dehydrated with graded alcohol, embedded in paraffin, and sectioned (thickness = 8 μm). Sections were then deparaffinized and rehydrated with xylene and graded alcohol. Hematoxylin and Eosin (H&E) staining were performed to evaluate the thickness of the skin samples; TUNEL assay was used to identify the apoptotic cells in the skin samples; and immunohistochemical staining of ki67 and CD86 (1:150 dilution, Beyotime) was applied to detect the proliferation of cells and inflammation in the skin samples.

### 2.12 Statistical analysis

Statistical analysis was carried out using IBM SPSS software (version 20). Results were shown as means ± standard error of the mean (SEM). Differences between groups were by one-way ANOVA followed significant and *p*-value less than 0.05 was considered as statistically significant.

## 3 Results

### 3.1 ELE reduces cell viability and induces apoptosis in M5-stimulated keratinocytes

**FIGURE 1:**
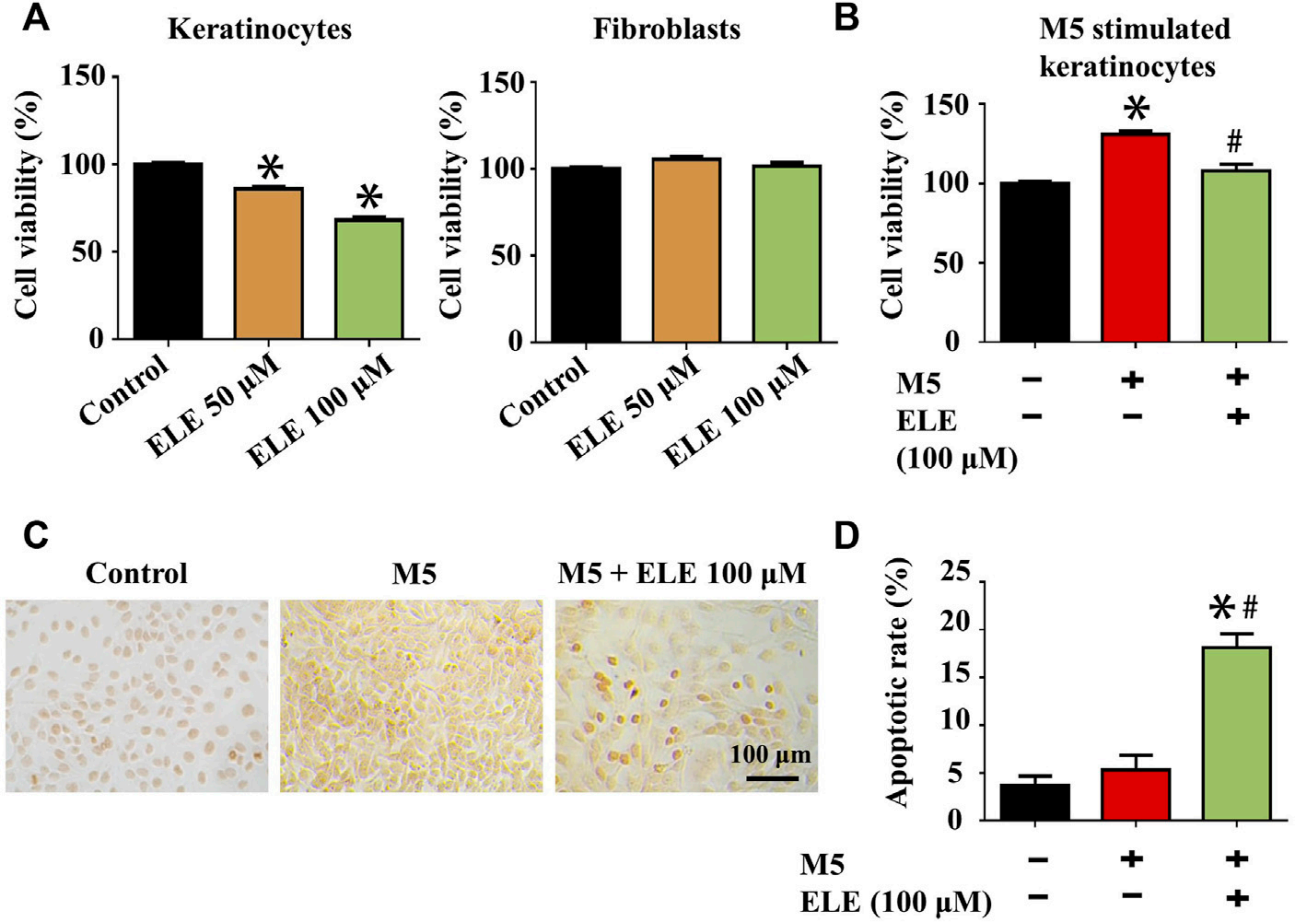
**(A)** Effects of ELE on the viability of keratinocytes and fibroblasts at 72 h. **(B)** Effects of ELE on the viability of M5-stimulated keratinocytes at 72 h **(C)** ELE-induced apoptosis cells (dark brown) detected in M5-stimulated keratinocytes using TUNEL assay. **(D)** Quantitative analysis of TUNEL assay. *p < 0.05 *versus* control, #*p* < 0.05 *versus* M5. Error bars indicate SEM (*n* = 3).

Effects of ELE on the viability of keratinocytes and fibroblasts were evaluated using CCK-8 assay. As shown in Figure 1A, ELE at 50 and 100 μM significantly attenuated keratinocytes viability by 14.08±1.4% and 31.83±1.63%, respectively. However, no inhibitory effects of ELE on fibroblasts were observed at the same concentrations, indicating that ELE is able to specifically inhibit the viability of keratinocytes. To further explore how ELE affects psoriatic keratinocytes *in vitro*, the effects of ELE on M5-stimulated keratinocytes were also investigated based on the previously published method (Gao et al., 2020). As illustrated in Figure 1B and C, ELE was found to inhibit cell viability and induce apoptosis in M5-stimulated keratinocytes. Quantitative analysis (Figure 1D) showed that ELE at 100 μM triggered 18.11±0.49% of cells undergoing apoptosis. Taken together, these data suggest that ELE specifically reduces the viability of keratinocytes without affecting the viability of dermal fibroblasts. In addition, ELE inhibits cell viability and induces apoptosis in psoriatic keratinocytes *in vitro*.

### 3.2 ELE-induced gene changes in M5-stimulated keratinocytes

**FIGURE 2:**
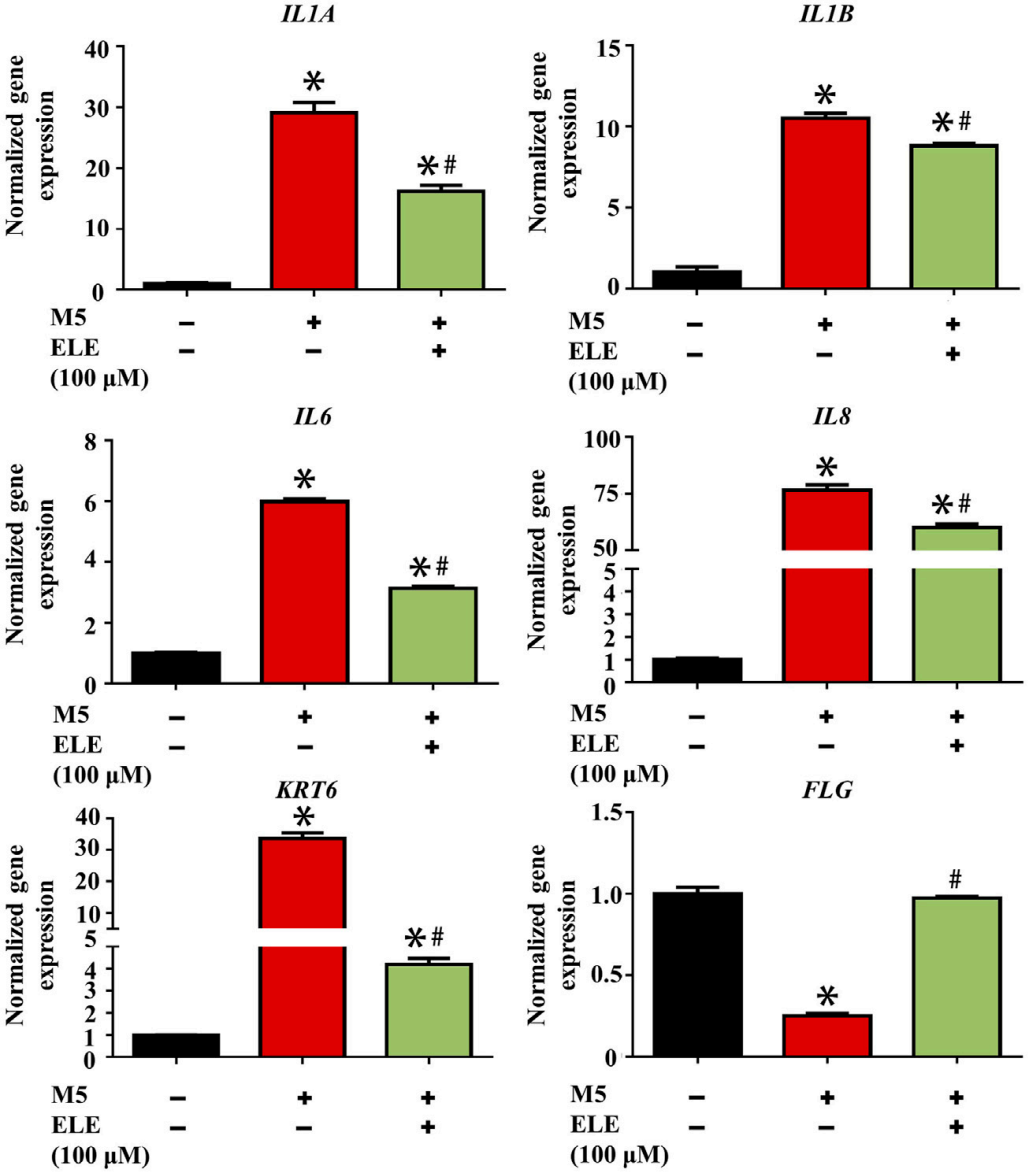
Effects of ELE on the expression of psoriasis-related genes in M5-stimulated keratinocytes. *p < 0.05 *versus* control, #*p* < 0.05 *versus* M5. Error bars indicate SEM (*n* = 3).

As shown in Figure 2, ELE was found to significantly attenuate M5-triggered up-regulation of *IL1A* (encoding IL-1α), *IL1B* (encoding IL-1β), *IL6* (encoding IL-6), and *IL8* (encoding IL-8). In addition, ELE also attenuated the M5-induced expression of *KRT6* (encoding keratin 6). Furthermore, M5 significantly reduced the expression of *FLG* (encoding filaggrin) in keratinocytes, while ELE improved the expression of FLG in M5-stimulated keratinocytes. Taken together, these data suggest that ELE inhibits M5-stimulated inflammation and proliferation in keratinocytes. It also protects the M5-induced damages to the barrier function of keratinocytes.

### 3.3 ELE indirectly attenuates keratinocytes inflammation by mediating M1-Mø

**FIGURE 3:**
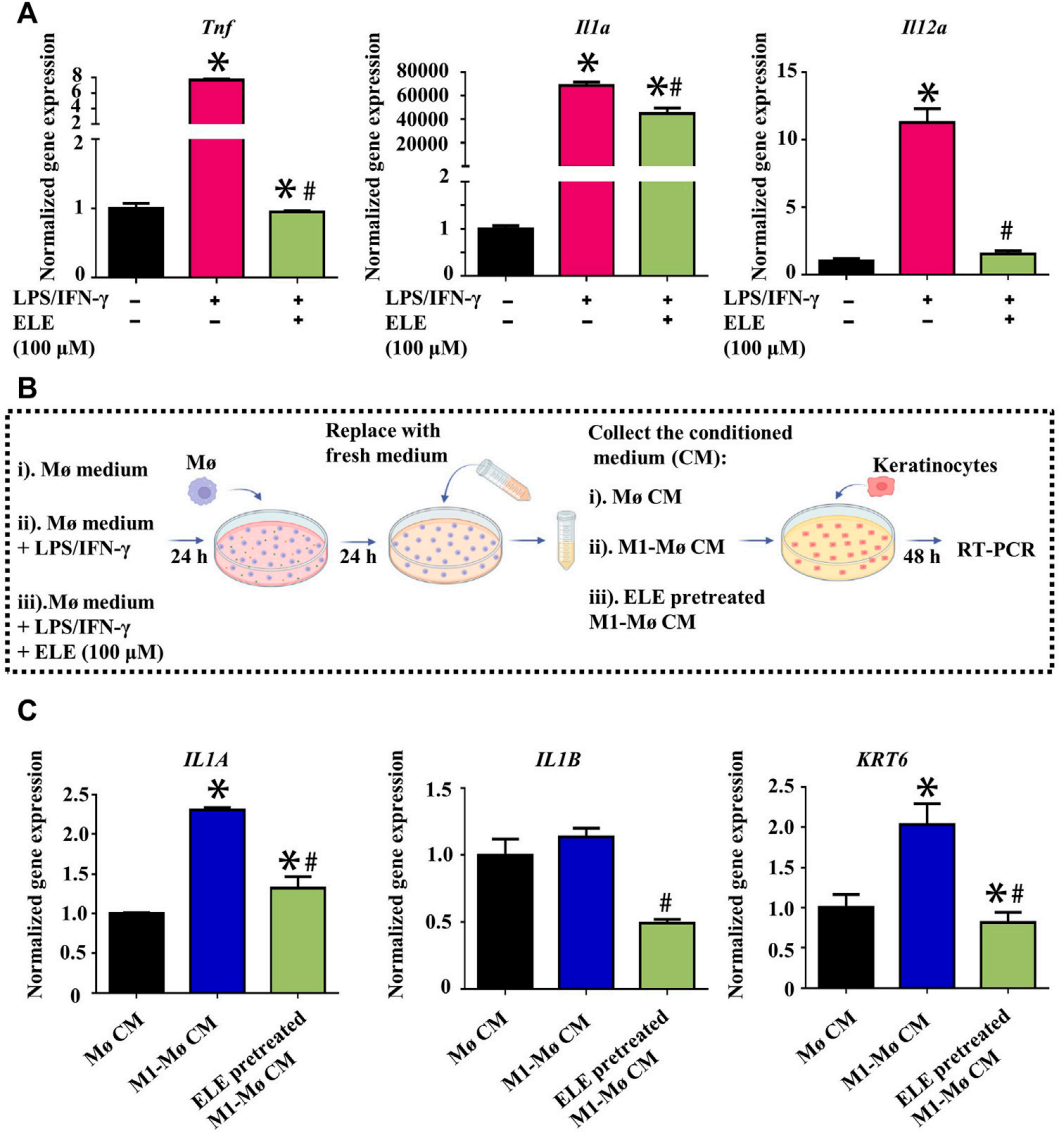
**(A)** Effects of ELE on the expression of Tnf, Il1a, and Il12a in M1-Mø. **(B)** Scheme illustration of the effects of Mø CM on keratinocytes. **(C)** Effects of M1-Mø and ELE-pretreated M1-Mø CM on the expression of IL1A, IL1B, and KRT6 in keratinocytes. *p < 0.05 *versus* control, #*p* < 0.05 *versus* LPS/IFN-γ or M1-Mø CM. Error bars indicate SEM (*n* = 3).

Elevated expression of M1-Mø is another key characteristic of psoriasis, it has been found that various inflammatory cytokines such as ILs and TNF-α secreted by M1-Mø contribute to psoriasis by triggering inflammation in keratinocytes (Kamata and Tada, 2022). We therefore stimulated Mø using LPS/INF-γ to obtain M1-Mø *in vitro* and then examined the effects of ELE on the gene expression of key inflammatory cytokines in M1-Mø. As shown in Figure 3A, ELE significantly down-regulated the expression of *Tnf* (encoding TNF-α), *Il1a* (encoding IL-1α), and *Il12a* (encoding IL-12A) in M1-Mø. In addition, we also investigated how the ELE-pretreated M1-Mø indirectly affects keratinocytes (Figure 3B). As demonstrated in Figure 3C, CM collected from M1-Mø significantly enhanced the expression of *IL1A* and *KRT6* in keratinocytes, while the expression of these two genes was significantly down-regulated after exposure to the ELE-pretreated M1-Mø CM. Although no up-regulation of *IL1B* was detected in keratinocytes treated with M1-Mø CM, ELE-pretreated M1-Mø CM significantly reduced the expression of *IL1B* in keratinocytes. These data indicate that ELE indirectly attenuates keratinocyte inflammation by inhibiting inflammatory cytokines released from M1-Mø.

### 3.4 Characterization of HA-ELE-MN

**FIGURE 4:**
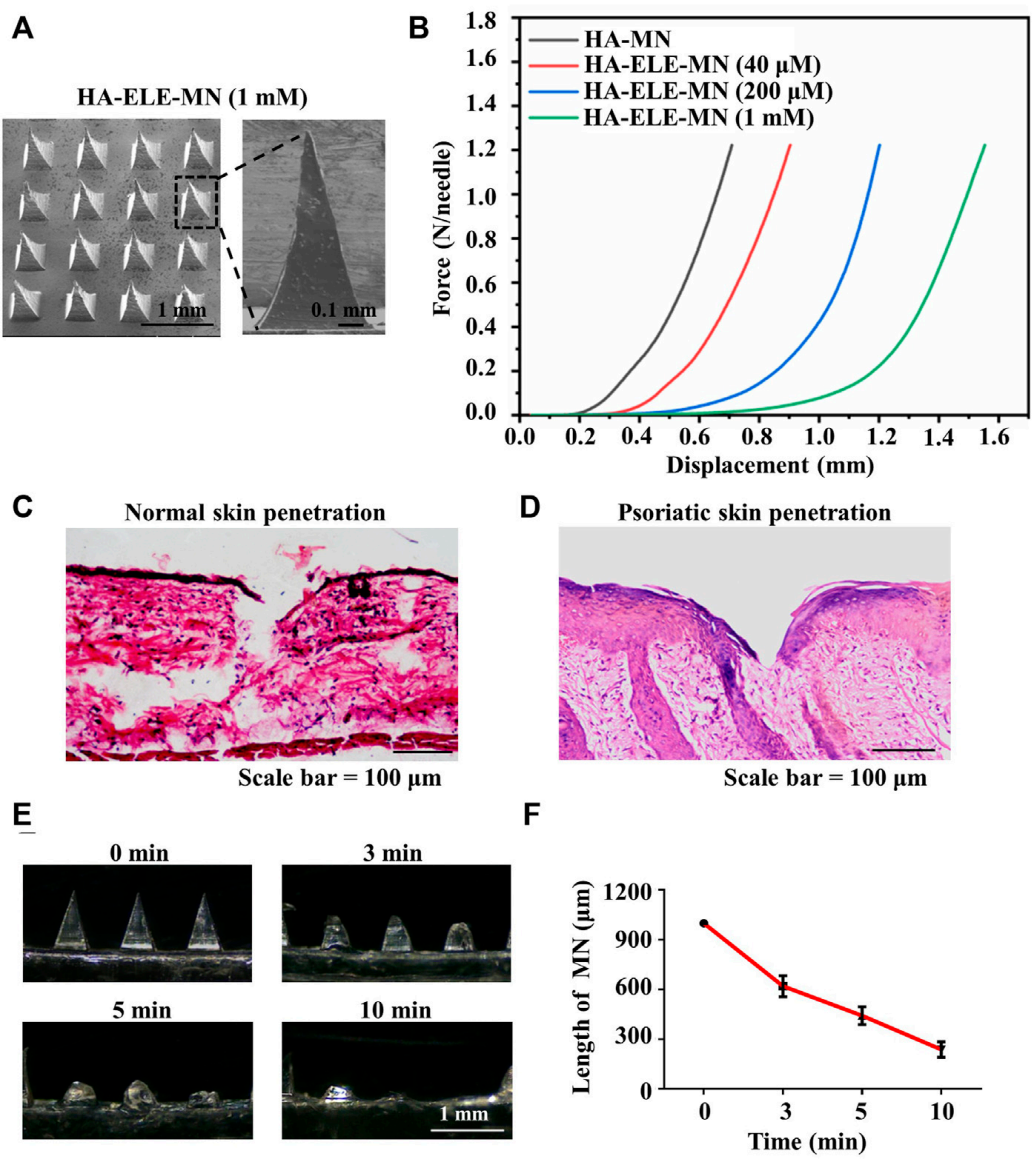
Characterizations of HA-ELE-MN (1mM). **(A)** Morphology of HA-ELE-MN (1mM) recorded using SEM. **(B)** Force-displacement curves of HA-ELE-MNs containing different concentrations of ELE. **(C)** H&E staining of normal back skin of BALB/c mice after application of HA-ELE-MN (1mM). **(D)** H&E staining of psoriasis skin of IMQ-induced psoriasis BALB/c mice after application of HA-ELE-MN (1mM). **(E)** Dissolution of HA-ELE-MN (1mM) in the skin of BALB/c mice. **(F)** Quantitative analysis of the speed of *in vivo* drug release by measuring the change of needle length.

As illustrated in Figure 4A, the needle arrays were intact and consecutive, and each single needle showed a triangular pyramidal shape. As demonstrated in Figure 4B, the stiffness of HA-ELE-MN dropped with the increase in ELE concentration, however, HA-ELE-MN at the highest ELE concentration (1 mM) was still strong enough to withstand a force of more than 1.2 N/needle without fracture. Previous studies reported that the minimum force required for skin penetration was less than 0.1 N/needle (Zhu et al., 2017), indicating that the HA-ELE-MN is capable to penetrate the skin. To further confirm this, we tested the penetrating ability of HA-ELE-MN (1 mM) on the back skin of BALB/c mice using H&E staining. As shown in Figure 4C, the HA-ELE-MN (1mM) successfully broke the epidermis and reached the dermis. Given that psoriasis skin normally has a thicker epidermis compared to normal skin, therefore the same test on the back skin of IMQ-induced psoriasis BALB/c mice was also performed. As can be seen in Figure 4D broken epidermis was observed in psoriasis skin after treated with HA-ELE-MN (1mM), indicating the HA-ELE-MN (1mM) is able to penetrate the psoriasis skin as well. Dissolution of HA-ELE-MN *in vivo* was tested by applying the HA-ELE-MN (1mM) on the back skin of BALB/c mice for 3, 5, and 10 min, respectively. As proved in Figure 4E and F, the needles containing ELE almost totally dissolved in the skin tissue within 10 min, indicating the fast drug release capability of the HA-ELE-MN (1mM).

### 3.5 HA-ELE-MN alleviates symptoms in IMQ-induced psoriasis model

**FIGURE 5:**
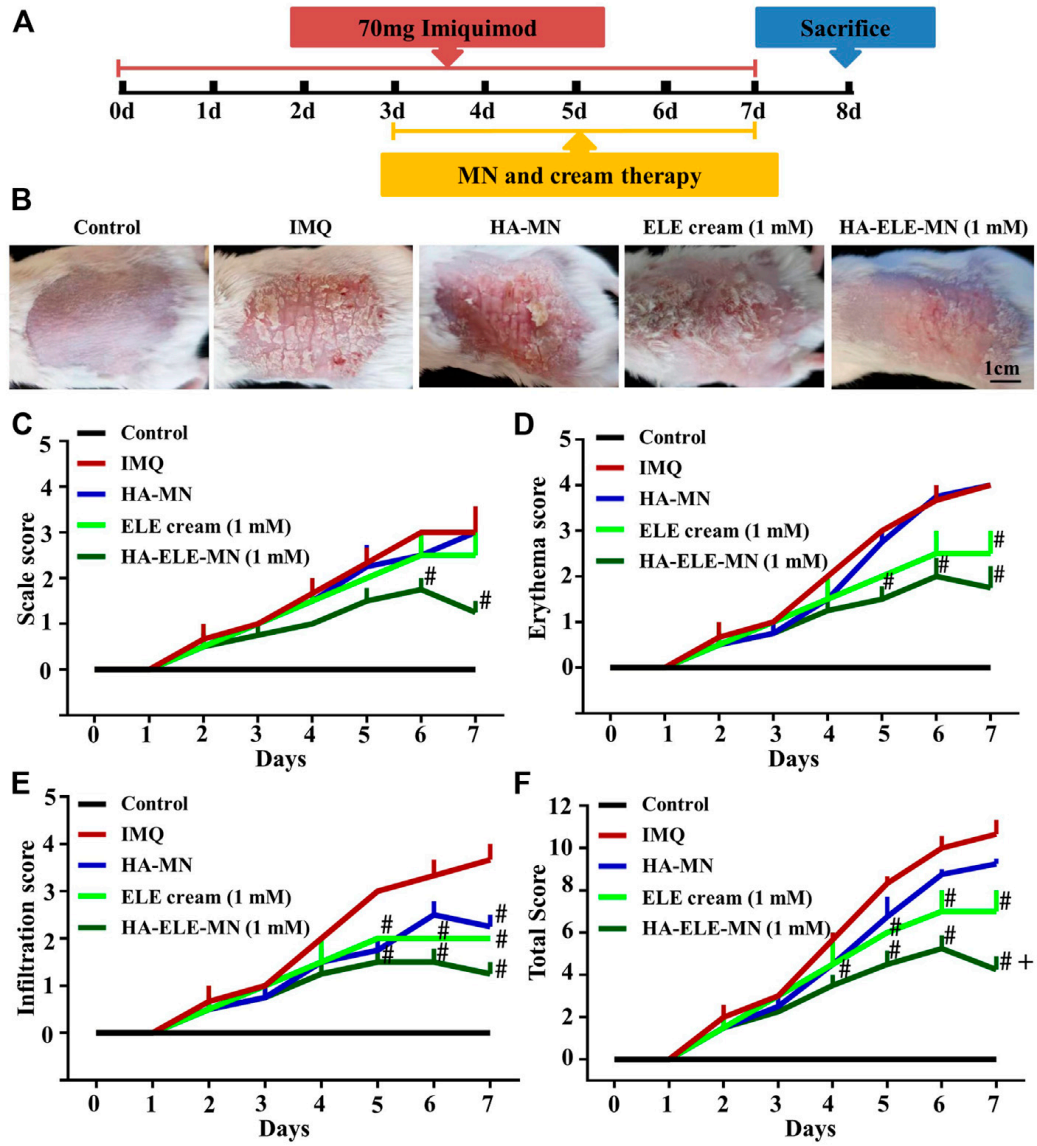
Mitigation of IMQ-induced psoriasis after exposure to HA-ELE-MN (1mM). **(A)** Schematic illustration of HA-ELE-MN (1mM) for the treatment of IMQ-induced psoriasis. **(B)** Representative photographs captured on day 7 (treated with IMQ for 7 days and exposed to ELE cream (1mM), HA-MN, and HA-ELE-MN (1mM) for 4 days). **(C–E)** PASI scores of scales, erythema, and infiltration. **(F)** Total PASI scores by pooling scales, erythema, and infiltration together. #*p* < 0.05 *versus* IMQ, +*p* < 0.05 *versus* ELE cream (1mM). Error bars indicate SEM (*n* = 3).

The therapeutic effects of HA-ELE-MN (1mM) were evaluated using PASI scores on an IMQ-induced psoriasis model. As can be seen in Figure 5B, symptoms including erythema, scaling, and infiltration were found to be alleviated in ELE cream (1mM), HA-MN, and HA-ELE-MN (1mM) groups compared to the IMQ group. HA-ELE-MN (1mM) showed better therapeutic outcomes compared to the HA-MN and ELE cream (1mM) groups. Based on the PASI scores, the scales (Figure 5C were significantly reduced in HA-ELE-MN (1mM) treated group compared to the IMQ group on day 6 and 7. Erythema (Figure 5D) on the back skin was significantly alleviated in HA-ELE-MN (1mM) treated group compared to the IMQ group on day 5, 6, and 7. ELE cream (1mM) was also found to attenuate the erythema compared to the IMQ group on day 7. Reduced infiltration (Figure 5E) was detected on day 5 in the HA-MN and HA-ELE-MN (1mM) groups, while attenuated infiltration was observed in the ELE cream (1mM) and HA-ELE-MN (1mM) group on day 6 compared to the IMQ group. Significantly down-regulated infiltration was found in HA-MN, ELE cream (1mM), and HA-ELE-MN (1mM) groups at day 7 compared to the IMQ group. Taken together, the total PASI scores shown in Figure 5F suggest that both ELE cream (1mM) and HA-ELE-MN (1mM) possess therapeutic effects on psoriasis, and HA-ELE-MN (1mM) generates better therapeutic outcomes compared to ELE cream (1mM).

### 3.6 HA-ELE-MN reduces epidermal thickness, induces apoptosis, inhibits cell proliferation, and attenuates M1-Mø infiltration in the IMQ-psoriasis model

**FIGURE 6:**
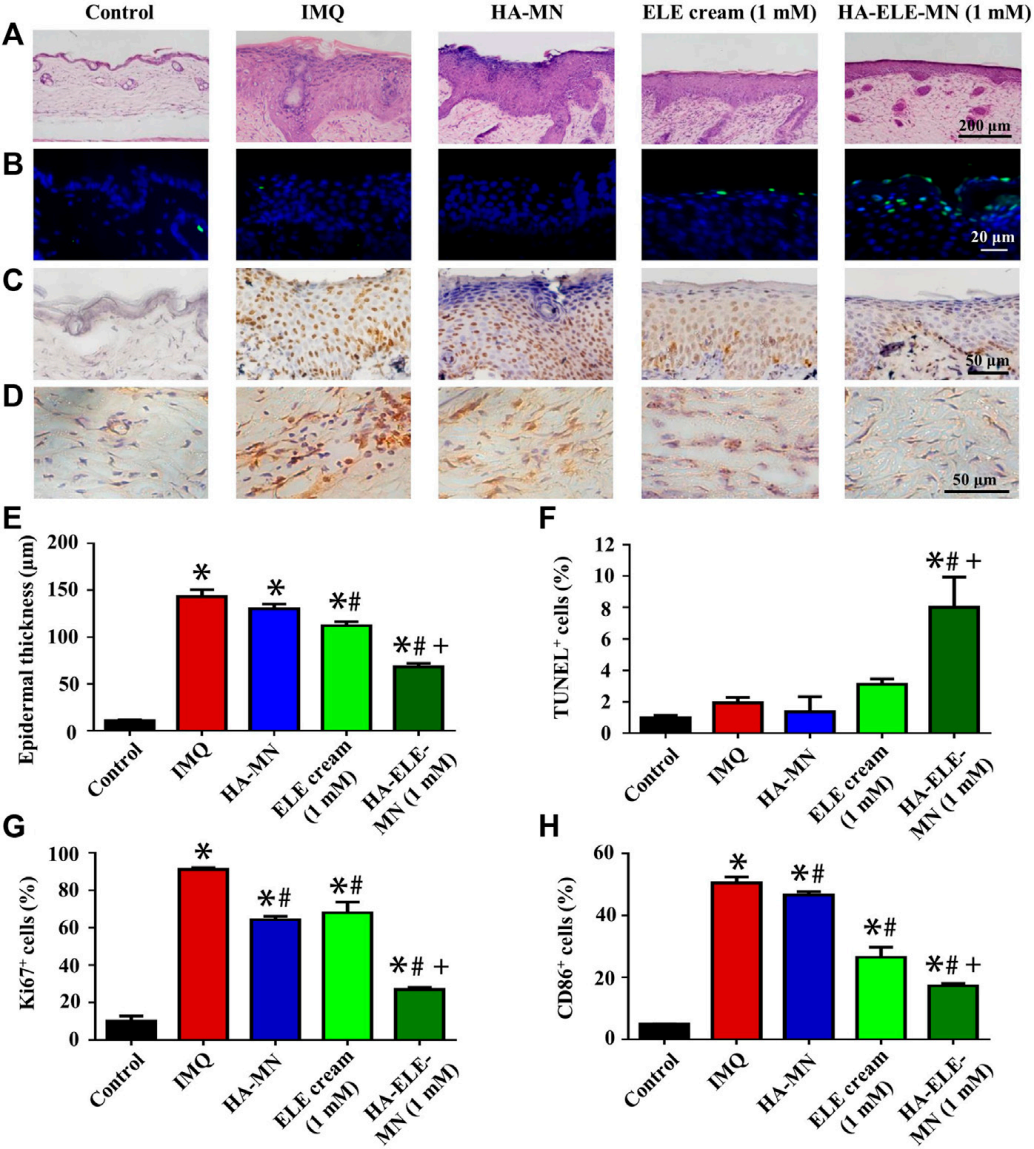
Histopathological analysis of skin samples in IMQ-induced psoriasis model after treated with HA-ELE-MN (1mM). **(A)** H&E staining for the assessment of epidermal thickness. **(B)** TUNEL staining. Green indicates apoptotic cells, blue indicates cell nucleus. **(C–D)** Immunohistochemical staining of ki67 and CD86. **(E–H)** Quantitative analysis of the epidermal thickness, TUNEL positive (apoptotic) cells, ki67^+^, and CD86^+^ cells. *p < 0.05 *versus* control, #*p* < 0.05 *versus* IMQ, +*p* < 0.05 *versus* ELE cream. Error bars indicate SEM (n = 3).

H&E staining was performed to assess the effects of HA-ELE-MN (1mM) on the epidermal thickness in the IMQ-induced psoriasis model (Figure 6A and E). It can be seen that HA-MN containing no ELE had no effect on the epidermal thickness compared to the IMQ group, however, both ELE cream (1mM) and HA-ELE-MN (1mM) groups significantly reduced the epidermal thickness compared to the IMQ group. In addition, the epidermal thickness in HA-ELE-MN (1mM) treated group was found to be significantly thinner than that in the ELE cream (1mM) group, suggesting a better therapeutic outcome by delivering ELE using MN.

TUNEL assay was performed to identify the apoptotic cells triggered by HA-ELE-MN (1mM) *in vivo*. As exhibited in Figure 6B and F, an increased number of apoptotic cells was observed in the ELE cream (1mM) group compared to the IMQ and HA-MN groups, however, no statistical differences were obtained in the quantitative analysis, suggesting the apoptosis-inducing ability of ELE is limited by the formulation. In contrast, a significantly increased number of apoptotic cells was detected in the HA-ELE-MN (1mM) group compared to all other groups.

In addition, increased expression of ki67 in the epidermis reflects enhanced proliferation of keratinocytes. It can be seen in Figure 6C and G that the expression of ki67 was significantly down-regulated after treated with HA-MN, ELE cream (1mM), and HA-ELE-MN (1mM) compared to the IMQ group. HA-ELE-MN (1mM) group showed higher inhibitory effects on the expression of ki67 compared to the ELE cream (1mM) group.

Furthermore, the effects of HA-ELE-MN (1mM) on the infiltration of M1-Mø in the dermis were examined by detecting CD86^+^ cells (Figure 6D and H), as CD86 is one of the commonly used biomarkers for M1-Mø. It can be seen that the number of CD86^+^ cells was significantly attenuated in HA-MN, ELE cream (1mM), and HA-ELE-MN (1mM) groups compared to the IMQ group. HA-ELE-MN (1mM) group exhibited higher inhibitory effects on the number of CD86^+^ cells compared to the ELE cream (1mM) group.

Taken together, these data demonstrate that ELE delivered using MN has better therapeutic effects compared to the cream formulation. The therapeutic effects of HA-ELE-MN (1mM) result from the apoptosis-inducing, proliferation-inhibiting, and inflammation-relieving properties of ELE.

## 4 Discussion

Psoriasis is a chronic inflammatory disease that involves the participation of keratinocytes, Mø, and other inflammatory cells (Dainichi et al., 2018). Although there are various therapies targeting different pathological processes of psoriasis, none of them have achieved satisfactory outcomes. Based on the biological activities of ELE, we proposed a psoriasis treatment strategy of using one single molecule to simultaneously regulate both keratinocytes and M1-Mø. Our results showed that ELE is able to alleviate psoriasis by simultaneously inducing apoptosis in keratinocytes, inhibiting keratinocytes inflammation, and suppressing the expression of inflammatory cytokines secreted by M1-Mø.

Inducing apoptosis in keratinocytes is one of the most important modalities for psoriasis treatment. The major issue of current apoptosis-inducing treatments, such as methotrexate and phototherapy, is their toxicity. It is therefore important to minimize the toxicity while maintaining the apoptosis-inducing ability. ELE has been previously reported to induce apoptosis in various cancer cell lines, including SiHa cervical squamous carcinoma cells and NCI-H1975 human lung adenocarcinoma cells (Wang et al., 2018; Wang et al., 2021). Due to its excellent biosafety, ELE injection and ELE oral emulsion have been approved as one of the Class II antitumor drugs (Zhai et al., 2018). However, the effects of ELE on psoriasis keratinocytes have not been investigated. In this study, we first examined the effects of ELE on keratinocytes as well as fibroblasts. The results showed that ELE at concentrations below 100 μM significantly inhibits the viability of keratinocytes without affecting fibroblast viability, indicating that ELE has low toxicity. To further investigate the role of ELE on psoriasis keratinocytes, we constructed an *in vitro* psoriasis model using M5-stimulated keratinocytes and detected the effects of ELE on these cells. Our data showed that ELE significantly inhibits cell viability and induces apoptosis in M5-stimulated keratinocytes. Data from animal experiments also indicated that HA-ELE-MN (1mM) inhibits keratinocytes proliferation (ki67^+^) and induces keratinocytes apoptosis (TUNEL^+^) in the IMQ-induced psoriatic mice model. These results demonstrate that ELE alleviates psoriasis by inducing apoptosis of keratinocytes.

In addition, the exaggerated inflammation in keratinocytes is another key reason for psoriasis (Ni and Lai, 2020). Studies have demonstrated that suppression of keratinocyte inflammation significantly alleviates psoriasis (Zuo et al., 2022). Previous studies have reported that ELE down-regulates the expression of TNF-α and IL-6 in 7,12-dimethylbenz(a)anthracene (DMBA)/12-O-tetradecanoylphorbol-13-acetate (TPA)-induced inflammation in mouse skin, indicating the potential anti-inflammatory activity of ELE (Hu et al., 2020). However, the effects of ELE on the inflammation of psoriatic keratinocytes have not been validated. It has been proven that the over-expression of interleukins in keratinocytes is associated with psoriasis, and inhibiting the expression of interleukins significantly relieves psoriasis (Tsai and Tsai, 2017). We therefore examined the effects of ELE on the inflammation of M5-stimulated keratinocytes. Our results showed that ELE significantly inhibits the expression of *IL1A*, *IL1B*, *IL6*, and *IL8* in M5-stimulated keratinocytes, demonstrating that ELE suppresses the inflammatory responses of psoriasis keratinocytes by attenuating the expression of various interleukins. Interestingly, we also found that ELE is able to decrease the expression of *KRT6* and promote the expression of *FLG* in M5-stimulated keratinocytes. It has been proven that the up-regulation of keratin 6/16/17 stimulates the proliferation, adhesion, and migration of keratinocytes (Zhang et al., 2019). In addition, previous studies also found that psoriasis is often accompanied by a decreased expression of *FLG*, suggesting that impaired epidermal barrier function is associated with psoriasis (Gutowska-Owsiak et al., 2012). Therefore, our results not only demonstrate that ELE possesses anti-inflammatory effects on psoriatic keratinocytes by attenuating the expression of interleukins, but also inhibits psoriatic keratinocytes proliferation by down-regulating *KRT6* and helps to maintain the barrier function of keratinocytes by stimulating the expression of *FLG*.

Furthermore, the infiltration of M1-Mø is another key characteristic of psoriasis. Previous studies have shown that the infiltration of M1-Mø greatly elevates the expression of inflammatory cytokines such as interleukins and TNF-α, which triggers psoriasis by stimulating the proliferation and inflammation of keratinocytes (Kamata and Tada, 2022). To date, little is known about the regulatory effects of ELE on Mø. A recent study reported that ELE inhibits the over-expression of IL-1 in LPS-stimulated Mø, suggesting the regulatory roles of ELE on M1-Mø (An et al., 2017). However, whether the regulatory effects of ELE on Mø help to relieve psoriasis is unknown. Our results showed that ELE significantly attenuates the expression of *Tnf*, *Il1a*, and *Il12a* in M1-Mø. Data from animal experiments also showed that HA-ELE-MN (1mM) significantly reduces the number of CD86^+^ cells in the dermis. These results demonstrated that ELE inhibits the infiltration and the expression of key inflammatory cytokines of M1-Mø. In addition, to further reveal how ELE alleviates psoriasis *via* the regulation of M1-Mø, we also examined the effects of ELE-pretreated M1-Mø CM on keratinocytes. Our results showed that ELE-pretreated M1-Mø CM significantly reduces the expression of *IL1A*, *IL1B*, and *KRT6* in keratinocytes, suggesting that ELE also indirectly inhibits the inflammation and proliferation of keratinocytes by regulating M1-Mø.

As mentioned above, inducing apoptosis in keratinocytes, inhibiting keratinocyte inflammation, or suppressing the expression of inflammatory cytokines from M1-Mø is able to alleviate psoriasis, however, therapies targeting one single pathological process fail to generate satisfactory outcomes. To address this, other studies also explored combination therapies for the treatment of psoriasis. For example, An et al. (2017) investigated a combination strategy using methotrexate and acitretin, in which methotrexate triggers apoptosis in keratinocytes while acitretin inhibits the infiltration of inflammatory cells. The results showed that this combination strategy generates better therapeutic outcomes compared to the application of methotrexate or acitretin alone (An et al., 2017). Similarly, Gold et al. (2021) explored the combination of calcipotriol and betamethasone dipropionate foam for psoriasis treatment and found that the therapeutic effects are significantly improved compared to the application of calcipotriol or betamethasone dipropionate alone (Stein Gold et al., 2021). Although these combination therapies have been demonstrated to provide better therapeutic outcomes than monotherapy, it makes the pharmacological mechanisms more complicated and the side effects are harder to be predicted. Based on the diverse biological activities of ELE, we proposed a psoriasis treatment strategy by using one molecule to simultaneously regulate keratinocytes and Mø. Our results demonstrated that ELE not only directly induces apoptosis and inhibits inflammation in psoriatic keratinocytes, but also indirectly attenuates keratinocytes inflammation and proliferation by suppressing the expression of key inflammatory cytokines secreted from M1-Mø. These findings confirm the feasibility of our proposed strategy for psoriasis treatment.

Given that the epidermis of psoriatic skin is dramatically enhanced compared to normal skin, it is therefore important to guarantee effective drug delivery for psoriasis treatment. Recent studies have shown that the use of MN technology for the delivery of anti-psoriasis drugs is a promising research direction (Shravanth et al., 2021). Based on its excellent biocompatibility, HA is widely used in the design of MNs. For example, Du et al. (2019) prepared an HA-MN loaded with methotrexate and found that this HA-methotrexate-MN generates better therapeutic effects on psoriasis compared to the oral application of methotrexate (Du et al., 2019). Based on this, we fabricated an HA-MN loaded with ELE and tested its therapeutic effects on psoriasis treatment using an IMQ-induced psoriatic mice model. Our results showed that HA-ELE-MN (1mM) generates better therapeutic outcomes compared to the ELE cream (1mM). These findings once again demonstrate the advantages of MN technology for psoriasis treatment.

## 5 Conclusion

**FIGURE 7:**
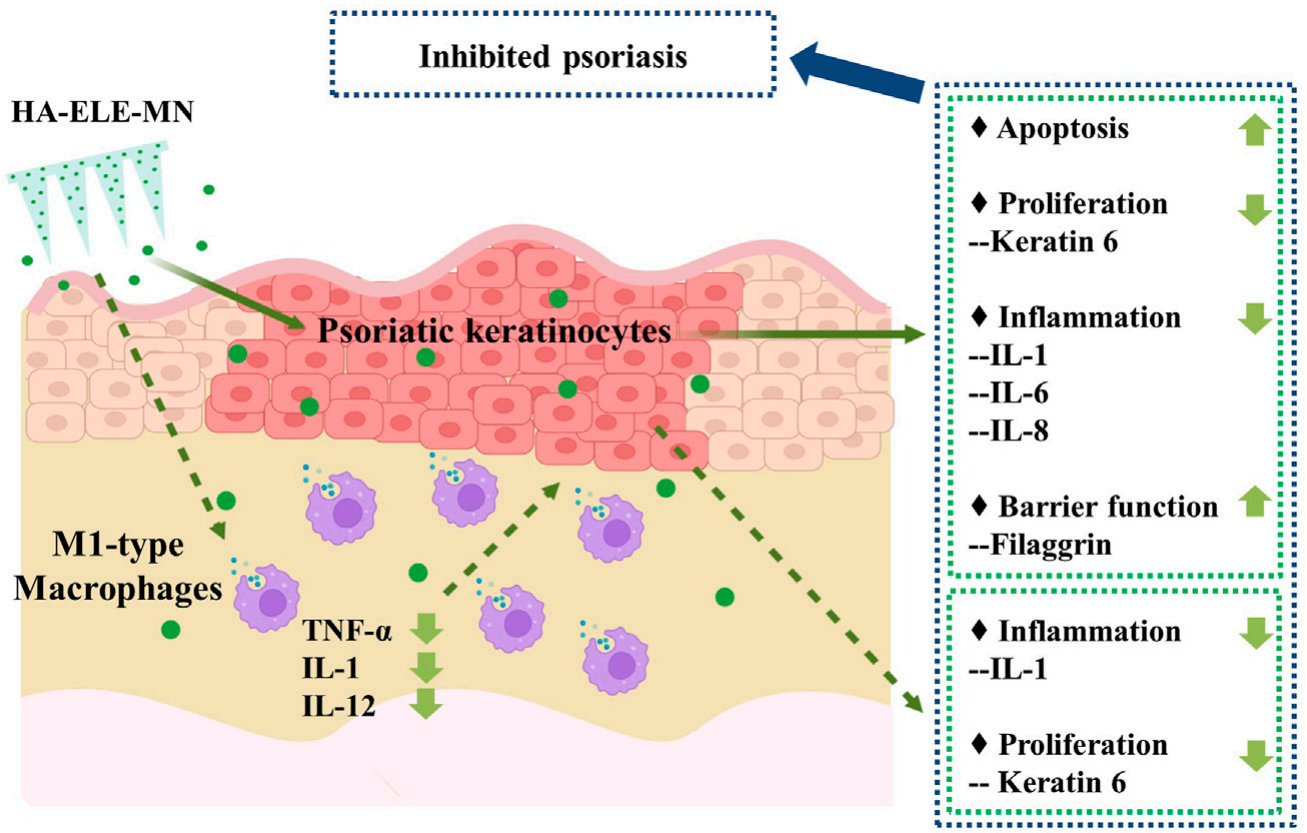
Potential mechanisms of ELE on psoriasis treatment. ELE directly induces apoptosis in psoriatic keratinocytes and inhibits psoriatic keratinocyte inflammation by down-regulating the expression of IL-1, IL-6, and IL-8. ELE may also reduce psoriatic keratinocyte proliferation by decreasing the expression of keratin 6 and help to maintain the barrier function of keratinocytes by stimulating the expression of filaggrin. In addition, ELE attenuates the expression of TNF-α, IL-1, and IL-12 in M1-Mø, which indirectly suppresses the inflammation and proliferation of psoriatic keratinocytes by attenuating the expression of IL-1 and keratin 6.

In conclusion, this study, for the first time, explored the direct effects of ELE on psoriasis keratinocytes and whether ELE indirectly affects keratinocytes *via* the regulation of M1-Mø. Based on the activities of ELE in triggering apoptosis in psoriatic keratinocytes, inhibiting psoriatic keratinocytes inflammation, and suppressing the expression of inflammatory cytokines from M1-Mø, we proposed a psoriasis treatment strategy by using one single molecule to simultaneously regulate multiple pathological processes (Figure 7) and validated its feasibility using an IMQ-induced psoriatic mice model. Data generated from this study not only provide a potential novel anti-psoriasis drug but also offer new insights into the future design of anti-psoriasis drugs.

## Data Availability

The raw data supporting the conclusion of this article will be made available by the authors, without undue reservation.
